# Supplementary material for: Perturbation of base excision repair sensitizes breast cancer cells to APOBEC3 deaminase-mediated mutations
Source: eLife. 2020 Jan 6;9:e51605. doi: 10.7554/eLife.51605 (PMC6961979; doi:10.7554/eLife.51605)
Supplement: Figure 2—source data 1. — RT2Profiler PCR screening for 84 human DNA repair genes. [file elife-51605-fig2-data1.docx]

**Figure 2—** **source data 1**

| RT^2^ profiler PCR screening for 84 human DNA repair genes | | | | | |
| --- | --- | --- | --- | --- | --- |
| Position | Symbol | Replicate 1 | Replicate 2 | Replicate 3 | Replicate 4 |
| A01 | APEX1 | -3.2 | -1.8 | -1.5 | -1.7 |
| A02 | APEX2 | -3.7 | -2.5 | -2.1 | -1.7 |
| A03 | ATM | 1.6 | 1.6 | 1.8 | 1.6 |
| A04 | ATR | -1.3 | 2.4 | 1.7 | 2.0 |
| A05 | ATXN3 | -2.0 | -2.1 | -1.4 | -1.5 |
| A06 | BRCA1 | -2.6 | 1.2 | 2.1 | 2.2 |
| A07 | BRCA2 | -4.0 | -3.0 | -1.1 | -1.2 |
| A08 | BRIP1 | -4.9 | -1.7 | -1.2 | -1.1 |
| A09 | CCNH | -1.1 | 1.3 | 1.2 | 1.3 |
| A10 | CCNO | -10.8 | -9.3 | -11.7 | -6.7 |
| A11 | CDK7 | 1.1 | 1.7 | 1.0 | -1.1 |
| A12 | DDB1 | -1.1 | 1.1 | 1.1 | 1.0 |
| B01 | DDB2 | -1.9 | -1.3 | 1.2 | 1.0 |
| B02 | DMC1 | -71.3 | -21.8 | -12.3 | -101.7 |
| B03 | ERCC1 | 2.1 | 5.4 | 3.4 | 3.5 |
| B04 | ERCC2 | 3.7 | 7.7 | 2.9 | 3.8 |
| B05 | ERCC3 | -2.4 | -1.4 | -1.1 | -1.2 |
| B06 | ERCC4 | -2.9 | -3.9 | -3.4 | -1.0 |
| B07 | ERCC5 | -1.8 | -1.6 | -1.3 | -1.3 |
| B08 | ERCC6 | 1.6 | 2.4 | -1.2 | 1.8 |
| B09 | ERCC8 | -1.2 | 1.5 | 3.1 | 1.3 |
| B10 | EXO1 | -18.8 | -10.4 | -2.4 | -3.1 |
| B11 | FEN1 | -3.9 | -2.8 | -1.2 | 1.0 |
| B12 | LIG1 | -7.1 | -5.6 | -5.5 | -2.1 |
| C01 | LIG3 | -2.2 | -1.3 | -2.0 | -1.7 |
| C02 | LIG4 | 1.6 | 2.1 | 4.3 | 2.8 |
| C03 | MGMT | -308.8 | -109.2 | -126.8 | -207.0 |
| C04 | MLH1 | -1.9 | -1.1 | 1.1 | -1.3 |
| C05 | MLH3 | -3.9 | -2.4 | -2.5 | -3.2 |
| C06 | MMS19 | -1.7 | 1.0 | -1.4 | -1.3 |
| C07 | MPG | -1.2 | -1.0 | -1.3 | -1.3 |
| C08 | MRE11A | -2.3 | -2.1 | -1.1 | -1.1 |
| C09 | MSH2 | -3.3 | -1.8 | -1.2 | -1.3 |
| C10 | MSH3 | -3.0 | -1.9 | -1.4 | -1.5 |
| C11 | MSH4 | -7.7 | -5.6 | -1.1 | 1.3 |
| C12 | MSH5 | -1.4 | -1.3 | -1.1 | 2.0 |
| D01 | MSH6 | -2.3 | 1.0 | -1.5 | -1.7 |
| D02 | MUTYH | -3.4 | -1.6 | 1.3 | -1.2 |
| D03 | NEIL1 | -50.8 | -19.1 | -36.1 | -27.2 |
| D04 | NEIL2 | 18.1 | 12.0 | 17.5 | 18.1 |
| D05 | NEIL3 | -3.9 | -2.5 | -1.5 | -1.0 |
| D06 | NTHL1 | -1.7 | 1.3 | -1.5 | -1.8 |
| D07 | OGG1 | -2.7 | -1.1 | 1.6 | 2.2 |
| D08 | PARP1 | -9.7 | -6.6 | -5.1 | -4.7 |
| D09 | PARP2 | -2.2 | -2.3 | -1.7 | -2.0 |
| D10 | PARP3 | 4.8 | 4.8 | 2.7 | 1.6 |
| D11 | PMS1 | -1.1 | -1.7 | -2.0 | -1.6 |
| D12 | PMS2 | -1.5 | 1.0 | -1.0 | -1.2 |
| E01 | PNKP | -1.8 | 1.3 | -1.3 | 1.0 |
| E02 | POLB | 1.2 | 2.1 | 1.3 | -1.0 |
| E03 | POLD3 | -5.1 | -3.0 | -2.1 | -1.7 |
| E04 | POLL | -2.4 | -2.2 | -2.0 | -1.1 |
| E05 | PRKDC | 1.2 | 3.4 | 2.2 | 2.0 |
| E06 | RAD18 | 1.0 | 1.2 | 4.6 | 1.5 |
| E07 | RAD21 | 1.2 | 1.3 | 1.8 | -1.0 |
| E08 | RAD23A | 1.4 | 3.1 | 2.2 | 1.7 |
| E09 | RAD23B | -1.5 | 2.4 | -1.1 | 1.6 |
| E10 | RAD50 | -2.0 | 2.3 | 1.9 | 1.8 |
| E11 | RAD51 | -2.6 | -3.8 | -1.7 | -2.2 |
| E12 | RAD51B | -4.6 | -4.8 | -3.3 | -2.6 |
| F01 | RAD51C | -1.4 | 1.4 | 1.5 | 1.4 |
| F02 | RAD51D | -5.0 | -1.5 | -1.4 | -1.2 |
| F03 | RAD52 | -4.1 | -1.9 | 1.2 | -1.3 |
| F04 | RAD54L | -3.5 | -1.7 | -2.3 | -1.0 |
| F05 | RFC1 | 1.1 | 1.3 | 1.4 | 1.4 |
| F06 | RPA1 | 1.3 | 2.1 | 2.6 | 2.2 |
| F07 | RPA3 | -1.7 | -2.3 | 1.0 | -1.1 |
| F08 | SLK | -1.0 | 2.2 | 2.2 | 1.5 |
| F09 | SMUG1 | -5.4 | -1.9 | -1.7 | -1.5 |
| F10 | TDG | -2.1 | -1.5 | -1.2 | -1.3 |
| F11 | TOP3A | -1.2 | -2.0 | -1.1 | 1.1 |
| F12 | TOP3B | -2.1 | -2.1 | -1.6 | 1.0 |
| G01 | TREX1 | 3.9 | 3.5 | 3.7 | 7.9 |
| G02 | UNG | -6.9 | -2.2 | -3.3 | -4.0 |
| G03 | XAB2 | -2.4 | -4.0 | -1.7 | -1.9 |
| G04 | XPA | 1.2 | -1.1 | -1.1 | 1.1 |
| G05 | XPC | -1.6 | 1.2 | 2.6 | 1.4 |
| G06 | XRCC1 | -1.7 | 1.4 | 1.2 | 1.2 |
| G07 | XRCC2 | -4.0 | -2.2 | 1.2 | 1.8 |
| G08 | XRCC3 | -6.0 | -6.4 | -5.3 | -1.3 |
| G09 | XRCC4 | -1.3 | 1.5 | 1.4 | 1.3 |
| G10 | XRCC5 | -2.5 | -1.6 | -1.4 | -1.6 |
| G11 | XRCC6 | -2.1 | -1.2 | 1.0 | -1.3 |
| G12 | XRCC6BP1 | -4.0 | -3.1 | -2.4 | -3.3 |
|  |  |  |  |  |  |
| * Numbers shown are fold change between Hs578T and MDA-MB-453 cells. | | | | | |
